# An Unexpected Airway Complication in a Male Patient with Goltz Syndrome

**DOI:** 10.1155/2016/4659891

**Published:** 2016-09-18

**Authors:** Sadie Smith, Kavita Gadhok, Dmitri Guvakov

**Affiliations:** ^1^Penn State Milton S. Hershey Medical Center, 500 University Drive, H187, Hershey, PA 17033, USA; ^2^Penn State Hershey Anesthesia, 500 University Drive, H187, Hershey, PA 17033, USA

## Abstract

Goltz syndrome, also known as focal dermal hypoplasia, is a rare X-linked dominant multisystem syndrome presenting with cutaneous, skeletal, dental ocular, central nervous system and soft tissue abnormalities. This case report discusses an adult male patient with Goltz syndrome that was noted to have large, papillomatous, hypopharyngeal lesions upon induction of general anesthesia. We highlight challenges with airway management intraoperatively and postoperatively in patients with Goltz syndrome. Our aim is to increase awareness of the potential airway complications associated with this genetic disorder and to provide suggestions for optimal perioperative management for patients afflicted with Goltz syndrome.

## 1. Introduction

Goltz-Gorlin syndrome (focal dermal hypoplasia or Goltz syndrome) not to be confused with Gorlin-Goltz syndrome (nevoid basal cell carcinoma), first described in 1962, is a highly variable condition that involves many organ systems of mesoectodermal origin [1]. The most common characteristic feature is thinning of the dermis resulting in subcutaneous fat herniation [2]. Areas that are frequently involved include skin (over 95% of reported cases), ocular, skeletal, craniofacial, and dental tissue [3]. Papillomas often appear in the skin, perioral, esophageal, perianal, genital, and ocular regions. There have been few reports of papillomas appearing in the upper airway (involving the tongue, tonsils, soft palate, pharynx, and larynx) [3–6].

This genetic disorder is inherited in an X-linked dominant manner and is lethal in males, unless it is expressed through somatic mosaicism [3]. Mutation of PORCN gene is associated with the disease and over 70 different mutations have been identified [7]. Over 90% of cases are female and 10% of cases are male [7]. A significant variation of disease expression and severity has been noted and is likely explained by functional mosaicism in female patients due to X chromosome inactivation and by somatic mosaicism in males [8].

This report describes the diagnosis of a laryngeal papilloma upon laryngoscopy, modified airway management, and the postoperative management of a male patient with Goltz syndrome.

## 2. Case Presentation

**Figure 1:**
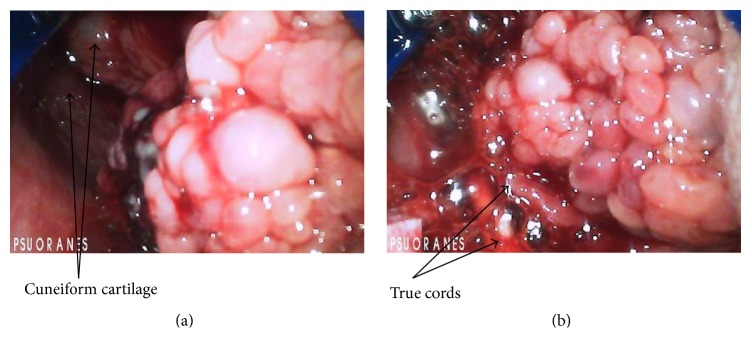
The large fungating mass coming from the base of the tongue obstructs the view of the epiglottis and vocal cords. The epiglottis is out of picture but would be close to the left upper corner of the picture, with the base of the tongue oriented towards the right border of the picture (a). After deeper insertion of the glidescope, the mass is seen hanging in the pharynx close to the true vocal cords, which are pulled to the right (b).

A 38-year-old Caucasian male with Goltz syndrome presented for elective surgery for removal of a left thigh papillomatous mass. He was born with webbed fingers and toes, as well as “splotchy skin.” He was diagnosed with Goltz syndrome with skin manifestations as an infant. In his late 20s, he started developing warts and skin tags over his body, and, later in his 30s, he started developing a papillomatous mass on his left inner thigh. His wife reported that the patient has been snoring for about 4 years; however, his snoring had significantly improved in the last 6 months. Other past medical history included obesity with a BMI of 43 and uncontrolled hypertension. Surgical history included a tonsillectomy as a teenager with no mention of anesthetic problems. He denied dysphagia and shortness of breath. Upon airway examination, he had a Mallampati score of 1 and full range of motion of his neck and mandible. The patient's physical examination was significant for a scattered, reticular rash across his chest, as well as a large papillomatous skin mass on his left inner thigh. Induction of anesthesia was accomplished with fentanyl, lidocaine, propofol, and rocuronium. A two-handed mask ventilation technique was required due to his body habitus, though no signs of airway obstruction were noted. Oxygen saturation during mask ventilation was maintained at 100%. Direct laryngoscopy was attempted with Macintosh 4 blade, a grade 2 laryngeal view was obtained, and a mass at the base of the tongue was visualized pulling the vocal cords to the right and obstructing the view of the vocal cords. A glidescope was then used in order to better visualize the mass. With optimal visualization, a friable, large, papillomatous mass was noted at the right base of the tongue (Figures 1(a) and 1(b)). The trachea was intubated with an 8 mm endotracheal tube. General anesthesia was maintained with sevoflurane, and the otolaryngology service was consulted intraoperatively for evaluation of the tongue mass. A small biopsy was taken, and then the surgical removal of the soft tissue mass on his thigh was performed. Upon completion of the thigh mass removal, the otolaryngology serviced returned for debridement of the tongue lesion to obtain a more clear view of the larynx and vocal cords. Upon completion of the debridement, the epiglottis and surrounding soft tissues were noted to be edematous. It was therefore decided to admit patient to the surgical intensive care unit and leave the trachea intubated overnight to safely allow the swelling to subside. After completion of three doses of dexamethasone 8 mg in the surgical intensive care unit, the trachea was uneventfully extubated and the patient was discharged home on postoperative day 2.

The pathological report for the debrided mass was interpreted as papillary lymphoid hyperplasia. The patient presented back to the operating room three weeks later for an elective removal of the remaining obstructing lesions [9]. He was electively extubated the next day in the surgical ICU, just as the previous procedure, to allow for airway edema recovery. At a follow-up clinic visit with otolaryngologist one week later, the patient was breathing well, with no areas of concern for residual or recurrent pharyngeal disease.

## 3. Discussion

Goltz syndrome is known to have variable manifestations involving the ectodermal and mesenchymal tissues with a broad phenotypical presentation. A prominent characteristic is the occurrence of papillomas of the skin and mucous membranes [6]. However, involvement of the laryngeal mucosa is very rare. Our patient had involvement of the tongue base, along with swelling of the epiglottis and hypopharyngeal tissues. Most patients with oral lesions develop symptoms of dysphagia, dyspnea, and/or snoring. However, our patient's snoring had improved in the last 6 months. It is possible that a portion of the laryngeal mass broke off and was swallowed, which would have improved his snoring and airway obstruction. The patient had surgery in the past under general anesthesia with no mention of papillomas. It has been reported that papillomas may develop throughout life and therefore can occur at any time [7]. Pathological examination of the laryngeal papillomas reveals hyperplastic, nonkeratinizing, squamous epithelium with sparse infiltration of lymphocytes as per Gordjani et al. [6], which is consistent with our patient's pathology report.

This syndrome is likely lethal in males unless expressed through postzygotic mosaicism [8]. Our patient is a very rare example of mosaicism and represents the wide range of manifestations of this disease in males.

One of the major anesthetic implications of Goltz syndrome is airway compromise from oral and laryngeal papillomas. As Holtzman describes, in an asymptomatic patient, after induction, moderate airway obstruction was noted and intubation was difficult due to friable obstructing lesions [5]. In our patient, we were able to visualize the glottic opening, and fortunately intubation was not tumultuous as it has been described in other case reports. It is therefore important to know this potential obstacle. In addition to a thorough history and physical examination, it may be worth considering a preoperative otolaryngologic exam and/or an awake flexible fiberoptic examination performed by the anesthesia team in patients with a known diagnosis of Goltz syndrome. This will evaluate the presence and/or extent of airway obstruction [10]. Spontaneous ventilation techniques that are performed in other cases of known airway lesions (i.e., tonsillar abscess and laryngeal carcinoma) may be suggested as an anesthetic technique in patients with Goltz syndrome [5]. It may be also beneficial to preoperatively counseling a patient with known Goltz syndrome about the possibility of an elective postoperative intensive care unit admission for airway edema recovery, in the event that an airway papilloma is encountered.

## 4. Conclusion

In patients with Goltz syndrome we strongly recommend performing a thorough airway exam, with possible preoperative otolaryngology consultation. In the event that Goltz syndrome papillomas are discovered in the airway, we recommend admitting patient to the ICU and delayed extubation until airway edema is resolved.

## References

[1] Smigiel R., Jakubiak A., Lombardi M. P. (2011). Co-occurrence of severe Goltz-Gorlin syndrome and pentalogy of Cantrell—case report and review of the literature. *American Journal of Medical Genetics Part A*.

[2] Thakkar S., Bharani S. (2013). A case report of focal dermal hypoplasia-Goltz syndrome. *Indian Dermatology Online Journal*.

[3] Wang L., Jin X., Zhao X. (2014). Focal dermal hypoplasia: updates. *Oral Diseases*.

[4] Kapoor S., Ghosh V., McGrath J. A., Kochar A. M., Kapoor H., Malik R. (2012). Novel mutation in a child with Goltz syndrome. *Indian Journal of Pediatrics*.

[5] Holzman R. S. (1991). Airway involvement and anesthetic management in Goltz's syndrome. *Journal of Clinical Anesthesia*.

[6] Gordjani N., Herdeg S., Ross U. H., Grimme H., Kleinschmidt M., Brandis M. (1999). Focal dermal hypoplasia (Goltz-Gorlin syndrome) associated with obstructive papillomatosis of the larynx and hypopharynx. *European Journal of Dermatology*.

[7] Ghoshal B., Lahiri S., Nandi D. (2012). A case of male Goltz syndrome. *Case Reports in Pediatrics*.

[8] Lasocki A. L., Stark Z., Orchard D. (2011). A case of mosaic Goltz syndrome (focal dermal hypoplasia) in a male patient. *Australasian Journal of Dermatology*.

[9] DiSalvo D. S., Oberman B. S., Warrick J. I., Goldenberg D. (2016). Pharyngeal presentation of Goltz syndrome: a case report with review of the literature. *Head and Neck Pathology*.

[10] Rhee K.-Y., Baek R.-M., Ahn K.-J. (2006). Airway management in a patient with focal dermal hypoplasia. *Anesthesia & Analgesia*.

